# Effect of Mild-to-Moderate Smoking on Viral Load, Cytokines, Oxidative Stress, and Cytochrome P450 Enzymes in HIV-Infected Individuals

**DOI:** 10.1371/journal.pone.0122402

**Published:** 2015-04-16

**Authors:** Anusha Ande, Carole McArthur, Leo Ayuk, Charles Awasom, Paul Ngang Achu, Annette Njinda, Namita Sinha, P. S. S. Rao, Marisela Agudelo, Anantha Ram Nookala, Stephen Simon, Anil Kumar, Santosh Kumar

**Affiliations:** 1 Division of Pharmacology and Toxicology, School of Pharmacy, University of Missouri-Kansas City, Kansas City, Missouri, United States of America; 2 Department of Oral and Craniofacial Science, School of Dentistry, University of Missouri-Kansas City, Kansas City, Missouri, United States of America; 3 Regional Hospital, Box 818, Bamenda, North West Province, Cameroon; 4 Mezam Polyclinic HIV/AIDS Treatment Center, Bamenda, Cameroon; 5 Department of Pharmaceutical Sciences, College of Pharmacy, University of Tennessee Health Science Center, Memphis, Tennessee, United States of America; 6 Department of Immunology, Herbert Wertheim College of Medicine, Florida International University, Miami, Florida, United States of America; 7 Department of Medical Informatics, School of Medicine, University of Missouri-Kansas City, Kansas City, Missouri, United States of America; Temple University School of Medicine, UNITED STATES

## Abstract

Mild-to-moderate tobacco smoking is highly prevalent in HIV-infected individuals, and is known to exacerbate HIV pathogenesis. The objective of this study was to determine the specific effects of mild-to-moderate smoking on viral load, cytokine production, and oxidative stress and cytochrome P450 (CYP) pathways in HIV-infected individuals who have not yet received antiretroviral therapy (ART). Thirty-two human subjects were recruited and assigned to four different cohorts as follows: a) HIV negative non-smokers, b) HIV positive non-smokers, c) HIV negative mild-to-moderate smokers, and d) HIV positive mild-to-moderate smokers. Patients were recruited in Cameroon, Africa using strict selection criteria to exclude patients not yet eligible for ART and not receiving conventional or traditional medications. Those with active tuberculosis, hepatitis B or with a history of substance abuse were also excluded. Our results showed an increase in the viral load in the plasma of HIV positive patients who were mild-to-moderate smokers compared to individuals who did not smoke. Furthermore, although we did not observe significant changes in the levels of most pro-inflammatory cytokines, the cytokine IL-8 and MCP-1 showed a significant decrease in the plasma of HIV-infected patients and smokers compared with HIV negative non-smokers. Importantly, HIV-infected individuals and smokers showed a significant increase in oxidative stress compared with HIV negative non-smoker subjects in both plasma and monocytes. To examine the possible pathways involved in increased oxidative stress and viral load, we determined the mRNA levels of several antioxidant and cytochrome P450 enzymes in monocytes. The results showed that the levels of most antioxidants are unaltered, suggesting their inability to counter oxidative stress. While CYP2A6 was induced in smokers, CYP3A4 was induced in HIV and HIV positive smokers compared with HIV negative non-smokers. Overall, the findings suggest a possible association of oxidative stress and perhaps CYP pathway with smoking-mediated increased viral load in HIV positive individuals.

## Introduction

The prevalence of mild-to-moderate tobacco smoking is approximately 3-fold higher in HIV-infected individuals (~60%) than in the general population (~ 20%) [1]. Smoking/nicotine has shown to enhance HIV-1 replication, especially in alveolar macrophages and microglia [2,3]. The alveolar macrophages obtained from smokers showed higher HIV p24 antigen levels following *in vitro* infection of HIV than those macrophages obtained from non-smokers [2]. Similarly, the pretreatment of HIV-infected microglial cells with nicotine increased HIV-1 expression in a concentration-dependent manner [4]. However, the specific effects of mild-to-moderate smoking on the components of HIV pathogenesis such as inflammatory cytokines and oxidative stress, is poorly studied.

Cytokines/chemokines play a crucial role in HIV pathogenesis by regulating HIV replication and immune responses [5,6]. Several studies have shown that cytokine dysregulation contributes to HIV disease progression [7,8]. For example, cytokines have shown to play an important role in development of viral latency and maintenance of latently infected CD4+ T cells during antiretroviral therapy (ART) [8]. Furthermore, the myriad of pro-inflammatory cytokines including tumor necrosis factor (TNF-α), interleukin-2 (IL-2), and IL-18, present in the plasma and lymphoid tissues, induce HIV replication even after prolonged ART. Similarly, oxidative stress is known to play a significant role in HIV-1 pathogenesis [9–11]. More specifically, oxidative stress has been linked to HIV-1 replication in monocytes/macrophages [9,12]. In addition, a report suggested the role of iron and oxidative stress in smoking-mediated HIV replication in alveolar macrophages [13]. Of importance, the pathway leading oxidative stress, especially in monocytes, in HIV-infected smokers is unknown.

The nicotine present in tobacco is predominantly metabolized by hepatic cytochrome P450 2A6 (CYP2A6) and lung specific CYP2A13 [14,15]. In smokers resulting reactive metabolites and oxidative stress, lead to cell toxicity, organ damage, and hepatic and pulmonary carcinomas [16,17]. Furthermore, our recent *in vitro* studies have also shown that acute nicotine exposure causes increased production of reactive oxygen species (ROS) through CYP2A6-mediated pathway in monocytic and astrocytic cell lines [18–20]. CYP3A4 metabolizes approximately 50% of commercially available drugs including ART in the liver [21] resulting in the production of ROS [22,23]. However, the functional role of CYP3A4 is unknown in monocytes, which is an important site of viral infection and major viral reservoir. Our previous studies have demonstrated predominant expression of CYP2A6 and CYP3A4 in monocytic cell lines [24]. In the current study, we sought to determine whether smoking increases viral replication in clinical samples of HIV-infected smokers. Furthermore, we sought to determine whether there is a relationship between viral load, cytokines/chemokines, oxidative stress, and CYP pathways in HIV-infected smokers.

## Materials and Methods

### Study Population

Thirty two human subjects were recruited and assigned to four different cohorts as follows: a) healthy HIV negative control subjects who reported that they were non-smokers (HIV negative non-smokers), b) HIV positive non-smokers, c) HIV negative mild-to-moderate smokers, and d) HIV positive mild-to-moderate smokers. Participants were recruited in Cameroon, Africa, from within a long standing reference population of more than 2000 known HIV positive subjects. The participants provided their written informed consent to participate in this study. The signed consent form from the participants was stored as hard copy as well as electronically on a password protected computer. The consent procedure was approved by Institutional Review Board (IRB) from the University of Missouri-Kansas City, Kansas City, MO and IRB/Institutional Ethic Committee (IEC) from the Provincial Regional Hospital, Ministry of Public Health, Bamenda, Cameroon. We performed power analysis using “Power and Precision” software by considering the magnitude of the known effects of smoking on HIV replication [4,13] and oxidative stress [18,19]. The results showed that 6–8 samples from each group would provide a power of≥0.80. Participants between the ages of 21–65 years were recruited, because individuals <13 years and >65 years of age generally have altered expression of metabolic (CYP) and to some extent antioxidant enzymes (AOEs) [25, 26]. For those individuals in the HIV negative non-smoker category, we recruited individuals who reported no history of “cough” and physical examination although some may have had malaria in the past (*Plasmodium falciparum* is indigenous in Cameroon). With respect to the HIV negative and positive smokers, mild-to-moderate smokers with a smoking history of≤20 pack years (a pack year is defined as smoking at least one pack a day for one year), were enrolled in the study. The documentation of smoking was determined by a HIV counselor and the phlebotomist following a personal interview. With respect to the HIV positive category, individuals with CD4+ counts <600 cells/μl were enrolled. Participants were recruited using the following strict exclusion criteria: 1) Pregnant or lactating women were excluded since they show increased metabolism of nicotine and cotinine [27] (smoking is rare among Cameroonian women); 2). Liver damage and lung disease since these alter the metabolism of tobacco constituents such as nicotine; 3) Individuals with other infectious diseases, such as documented malaria, tuberculosis, and hepatitis B since hepatitis and active TB are prevalent in HIV-infected population, and some of these diseases are known to interact with HIV [28]; 4) Individuals who were receiving ART. ART drugs or other medications are expected to interfere with tobacco constituents [16,29]. Sufficient numbers of HIV-infected individuals who were not already receiving ART or other medication were available among village populations in Cameroon, because medications or access to CD4 counting is frequently not available in these areas (WHO, Cameroon 2004–2005 report http://www.who.int/hiv/HIVCP_CMR.pdf). In order to qualify for free therapy provided by the Cameroonian Government, an adult must obtain a CD4 count and that result must be under 350cells/μl. The test to determine CD4 count is costly and not available in rural areas. Second, the local recommendation during the period of the study was to initiate therapy when the CD4 count was <350 cell/μl. 5) Individuals who consume other recreational substances of abuse, e.g. methamphetamine, cocaine, or marijuana. These drugs are expected to interfere with HIV pathogenesis and/or oxidative stress [30, 31]. Applying these inclusion and exclusion criteria was challenging but subjects were recruited upon personal interview, analysis of their history, and following clinical screening for HIV, malaria, and hepatitis B. The clinical screening tests for malaria and hepatitis were conducted using standard procedures as described [32] and patients with “cough” suggestive of TB received an AFB or were excluded.

### Determination of CD4 count and viral load

Whole blood was drawn in EDTA tubes from each subject who qualified for the study followed by confirmation of HIV status and a CD4 count by flow cytometry (Becton Dickenson, San Jose, CA). The viral load of HIV-infected individuals was determined in plasma using quantitative reverse transcriptase polymerase chain reaction (RTPCR) (Roche Amplicor System, Biocentric) at Pasteur Institute, Yaounde, Cameroon. The remaining sample of plasma from each patient was frozen immediately and shipped on dry ice to the University of Missouri-Kansas City for analysis of cytokines and determination of markers of oxidative stress and CYP enzymes.

### Preparation of peripheral blood mononuclear cells and monocytes

For the peripheral blood mononuclear cells (PBMC) isolation, gradient centrifugation technique was employed using ficoll hypaque plus. From the PBMCs, monocytes were isolated using dynabeads flowcomp human CD14 kit (Invitrogen, Grand Island, New York). The monocytes were immediately lysed using RLT buffer provided in the All prep DNA/RNA/Protein kit (Qiagen, Valencia, CA). The lysed samples were frozen and shipped to the University of Missouri-Kansas City for further studies.

### Cigarette smoke condensate (CSC) treatment and HIV replication in primary macrophages

Primary monocytes were differentiated into macrophages and infected with HIV-1, prior to treatment with CSC (Murty Pharmaceuticals, KY), using previously reported protocol [33, 34]. Briefly, PBMCs were plated in T-75 flask and non-adherent cells were removed after 1 h at 37°C. Cells were cultured for 6–8 days in media containing human mCSF to facilitate macrophage differentiation. Mature macrophages were collected and activated by treating with polybrene (2 μg/ml) for 30 min before HIV infection. Macrophages were infected with TCID_50_ of HIV-1 for 2 h and cultured in 6-well plates (1 million/well) for 6 days. Cell supernatant was collected on days 0, 3, and 6 to analyze p24 levels. Starting day 7, HIV-infected macrophages were treated, once daily, with 25 ug/ml CSC or equivalent amount of DMSO. During the treatment period, one-half milliliters of fresh media was added daily to each well to avoid cytotoxicity. Supernatant collected on day 11 (24 h after last treatment) was analyzed for viral load using the HIV type 1 p24 antigen ELISA (ZeptoMetrix, NY).

### Multiplex cytokine assay

The protein levels of various pro-inflammatory cytokines/chemokines; RANTES, IL-6, IL-8, monocyte chemotactic protein-1 (MCP-1), IL1-β, and TNF-α were measured using multiplex cytokine assay kit (Bio-Rad, CA, USA) as per the manufacturer’s protocol. Briefly, plasma samples were centrifuged at 3000 g for 10 min and the supernatants were diluted with three volumes of sample diluent. Next, 50 μl of each sample and standards were mixed with magnetic beads and incubated on a shaker at room temperature for 30 min. The beads were washed and 25 μl of detection antibody was added to each well followed by incubation for 30 min at room temperature. Furthermore, the samples were washed and incubated with 50 μl streptavidin-PE conjugate for 10 min. Finally, 125 μl of the assay buffer was added and the samples were analyzed using Biorad Bioplex HTS (Bio-Rad, CA, USA). The concentrations of the cytokines were determined with Bio-plex manager 5 using 5-PL statistics using standard curve.

### Quantitation of 8-Hydroxy-2′-deoxyguanosine content

The concentration of 8-Hydroxy-2′-deoxyguanosine (8-OHdG) was determined in the plasma samples using oxiselect oxidative DNA damage ELISA kit (Cell Biolabs, San Diego, CA) according to manufacturer’s instructions. Briefly, after diluting the plasma 50:50, the samples along with the 8-OHdG standards provided in the kit were added to an 8-OHdG/BSA conjugate on a preabsorbed EIA plate. After incubation for an hour, an anti-8-OHdG primary antibody was added, followed by an HRP conjugated secondary antibody. After incubation with substrate solution for an hour, the reaction was terminated using the assay “stop solution” and the absorbance was measured at 450 nm using a plate spectrophotometer. The 8-OHdG content of the DNA in monocytes was estimated using EpiQuik 8-OHdG DNA Damage Quantification Direct Kit (Fluorometric) (Epigentek, Farmingdale, NY) following manufacturer’s instructions. It is a highly sensitive fluorometric technique to detect 8-OHdG by using as low as 300 ng of unmodified DNA. Initially, DNA is bound to the wells that have a high DNA binding affinity. Then the 8-OHdG present in the samples is detected by using capture and detection antibodies. The enhancer solution is used to enhance the signal followed by quantification by reading the plate in a fluorescence microplate reader equipped with an excitation filter of 530 nm and emission filter of 590 nm. The amount of 8-OHdG in each sample is calculated by using a standard curve generated from a series of standards.

### DNA/RNA/Protein Isolation

Isolation of DNA, RNA, and protein was done from the lysed samples using the All prep DNA/RNA/Protein QIAGEN Kit (Valencia, CA) by following the manufacturer’s instructions. Briefly, the lysed sample was homogenized and transferred to the DNA column and the flow through was transferred to RNA column for obtaining RNA. The flow through from RNA column was used to precipitate protein using APP buffer. The RNA and DNA were quantified using UV spectrophotometer by measuring their absorbance at 260nm. The protein was quantified using BCA protein assay kit (Thermo scientific, Rockford, IL). However, the quantity of protein from the some samples, especially HIV positive smokers and HIV positive nonsmokers, were very low and insufficient for complete analysis.

### Quantitative reverse transcriptase polymerase chain reaction (RTPCR)

Quantitative RTPCR was performed as described in our previous studies [18]. Briefly, 60 ng of RNA was reverse transcribed to cDNA and then amplified in an iCycler iQ system (Bio-Rad Laboratories, Hercules, CA) using a two-step TaqMan Gene Expression Kit (Applied Biosystems, Foster City, CA). We measured the gene expression levels of following CYPs and AOEs: CYP2A6 (Hs0071162 _ m1), CYP2E1 (Hs00559367_m1), CYP3A4 (HS00430021_m1), Catalase (Hs00156308 m1), SOD1 (Hs00533490 m1), and Nrf2 (Hs00232352_m1) by using the probes obtained from Applied biosystems. The relative fold expression was calculated using 2^-ΔΔCt^ method by employing glyceraldehyde 3-phosphate dehydrogenase (GAPDH) as the housekeeping gene.

### Statistical analysis

Demographic variables (age, sex ratio) were summarized using descriptive statistics. All outcome variables were summarized as the mean plus or minus the standard error. Comparisons among the four groups were conducted using one way ANOVA with no corrections for multiple comparisons. All tests were two-sided and results that were statistically borderline significant at p≤0.1 (#) and significant at p ≤0.05 (*) and ≤0.01 (**). A two-way ANOVA was done to determine whether they interact synergistically or additively in HIV positive smokers. All analyses were performed using IBM SPSS version 21.

## Results

### Characteristics of the study population

A total of 32 subjects were selected upon screening approximately 2000 HIV positive individuals attending the HIV clinic at the Regional Hospital, Mezam Polyclinic HIV/AIDS Treatment Center, or at surrounding village health centers. The recruitment was very difficult and time consuming owing to strict exclusion criteria, paucity of smokers who also do not also drink alcohol and are not yet receiving ART or other medications. Furthermore, smoking was rare among women in this region, especially in HIV positive population in which it is discouraged. The subjects with such inclusion criteria are nearly impossible to recruit in the USA, because HIV-infected individuals receive ART promptly after diagnosis and most receive other medications and/or abuse illegal drugs. The recruitment of subjects who were not receiving ART was critical to determine the specific effects of mild-to-moderate smoking in HIV-infected individuals without an interference by drugs on the results of the study. The number of individuals in each group (HIV negative non-smoker, HIV negative smoker, HIV positive non-smoker, and HIV positive smoker) along with the age-range, median age, and male/female ratio of all the subjects are presented in Table 1. The median age of all the groups was 35–45. The male/female ratio varied in each group, with relatively low ratio in HIV positive group but high ratio in all smokers because of the low incidence of smoking among women with the strict exclusion criteria. Our analysis showed that the subject-to-subject variation in CD4 counts, viral load, cytokine production, and oxidative stress was not significant on the basis of age and gender differences (data not shown). This analysis was performed using HIV negative non-smokers and HIV negative smokers, in which, the number of subjects were sufficient to perform such analysis.

**Table 1:** Demography and clinical outcomes (CD4) of the subjects.

Subjects		HIV negative non-smokers	HIV positive non-smoker	HIV negative smokers	HIV positive smokers
Number, age (years), male/female ratio	#	11	6	11	4
	Age range	20–64	23–42	31–60	38–57
	Median age	45	34.5	45.5	42
	Male/female	1.2	0.5	1.75	3
CD4 count (cells/μL)	Range	631–1086	256–551	500–1488	13–584
	Mean ± SE	865 ± 45	374 ± 43	1037 ± 100	387 ± 130

### Effect of smoking and HIV on CD4 and viral load in plasma

**Fig 1:**
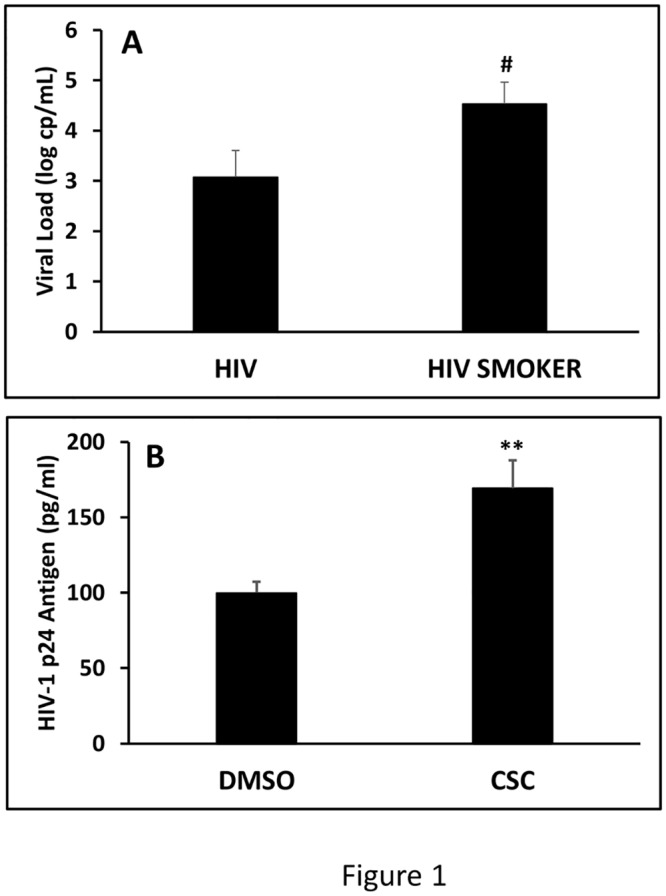
Determination of viral loads in HIV-infected subjects (A) and HIV-infected macrophages (B). The viral loads in the plasma of human subjects and macrophages were determined by analyzing HIV RNA using q-RTPCR and p24 using ELISA, respectively. The data for in vitro assay (B) represents mean of multiple analysis of p24 titer obtained from HIV-infected macrophages from three different donors. The p values (# and ** represent p≤0.1 and p≤0.01, respectively) are calculated using one way-ANOVA and presented in the graph.

The mean CD4 count (cells/μL) and mean viral load (log copies/mL) of each cohort are presented in Table 1 and Fig 1. As expected, the CD4 count was lower in HIV positive non-smokers (374 ± 43) and HIV positive smokers (387 ± 130) than HIV negative non-smokers (865 ± 45) or smokers (1037 ± 100). There was a significant increase in the CD4 count in HIV negative smokers compared with non-smokers (1037 ± 100 vs. 865 ± 45). Our results showed that the viral load is significantly increased in HIV positive smokers (4.5 ± 0.7) compared to the HIV positive non-smokers (3.1 ± 0.5) (Fig 1A). These results suggest that smoking is associated with increased viral replication.

### Effect of CSC on HIV replication in primary macrophages

To further confirm the increase in viral load in HIV-infected smokers compared with HIV-infected non-smokers, we treated HIV-infected primary macrophages with CSC for four days (once-daily). CSC constitutes the majority of the cigarette constituents and is an accepted in vitro substitute of smoking. The results showed that the amount of p24 antigen in cell free supernatant was found to be significantly higher in CSC treated HIV-infected macrophages compared to DMSO treated HIV-infected macrophages (Fig 1B).

### Effect of smoking and HIV on cytokine/chemokine production in plasma

**Fig 2:**
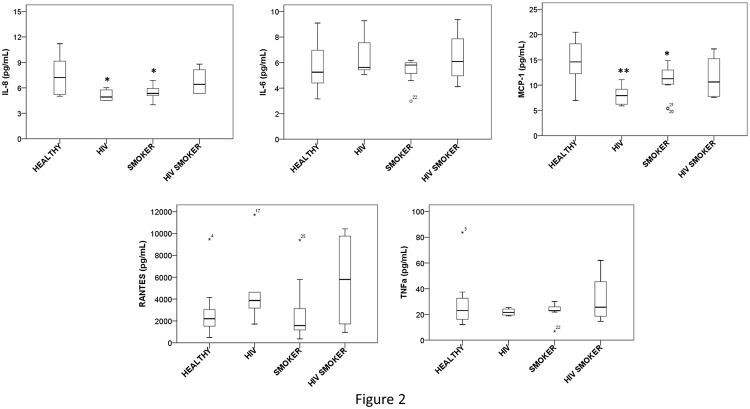
Box and whisker plots of cytokine/chemokine levels in plasma of HIV negative non-smokers (HEALTHY), HIV positive non-smokers (HIV), HIV negative smokers (SMOKER), and HIV positive smokers (HIV SMOKER) groups. The box represents the 25^th^-75^th^ quartile, the whiskers represent the range of values, the median is presented as a line inside the box, and the out of range values are presented as circles or stars above and below the whiskers. The p values (* and ** represent p≤0.05 and p≤0.01, respectively) are calculated using one way-ANOVA and presented above the bars in the graph. The concentrations of RANTES, IL-6, IL-8, MCP-1 and TNF-α were shown in the figure.

We determined the levels of various pro-inflammatory cytokines, IL-1β, IL-6, IL-8, RANTES, TNF-α and MCP-1 in all plasma samples. The levels of IL-1β were not detectable in any plasma sample within the detection limit of the assay (2 pg/mL). The concentrations of cytokines in each group showed significant subject-to-subject variations, and therefore, we presented these results in box plot as shown in Fig 2. The concentrations of most of the cytokines were in the range of 10–80 pg/mL, while the concentration of RANTES ranged from 500–12,000 pg/mL. The concentrations of MCP-1 and IL-8 were significantly lower in HIV positive non-smokers and HIV negative smokers and marginally lower in HIV positive smokers than HIV negative non-smokers (Fig 2). The concentration of RANTES was marginally higher in HIV positive non-smokers and smokers than HIV negative non-smokers and smokers. However, there were no significant differences in the levels of IL-6 and TNF-α between the test and control groups (Fig 2).

### Effect of smoking and HIV on oxidative stress in plasma and monocytes

**Fig 3:**
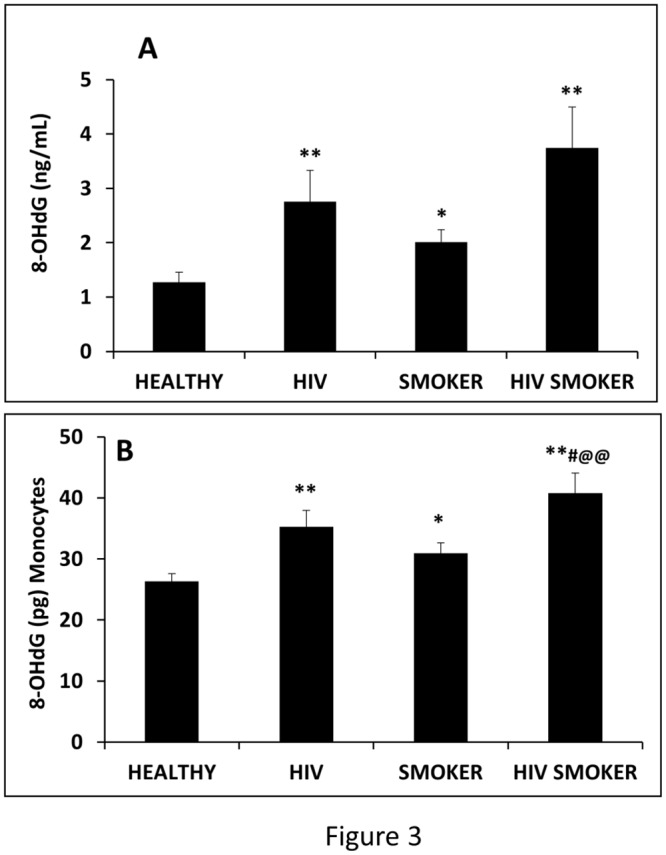
Measurement of oxidative stress in plasma and monocyte samples of HIV negative non-smokers (HEALTHY), HIV positive non-smokers (HIV), HIV negative smokers (SMOKER), and HIV positive smokers (HIV SMOKER) groups. The 8-OHdG contents are plotted as a bar graph for each category, and the p values ≤0.05 and ≤0.01 are represented as * and **, respectively. * represents the significance with respect to HEALTHY, # represents the significance with respect to HIV and @ represents the significance with respect to SMOKER in HIV SMOKER group.

To evaluate oxidative stress in clinical samples, we determined the oxidative DNA damage by measuring 8-OHdG content. The results showed that the oxidative damage is significantly increased in plasma from HIV negative smokers (2-fold), and HIV positive non-smokers (2.5-fold) groups compared with HIV negative non-smokers (Fig 3). Furthermore, there was an additive increase in the levels of oxidative damage in HIV positive smokers (4-fold) compared to HIV positive non-smokers and HIV negative smokers (two-way ANOVA for testing the interaction gave p = 0.39 suggesting the absence of synergy). Similarly, we determined the oxidative DNA damage in the monocytes of all the cohorts. Our results demonstrated an increase in 8-OHdG levels in HIV negative smokers (~30%) and HIV positive non-smokers (~40%) compared to HIV negative non-smokers (Fig 3). An additive increase was also observed in 8-OHdG levels in the DNA of HIV positive smokers (~75%) compared to HIV positive non-smokers and HIV negative smokers. Overall, the results suggest an increase in oxidative stress as a result of HIV infection and smoking.

### Effect of smoking and HIV on the expression of antioxidants in monocytes

**Fig 4:**
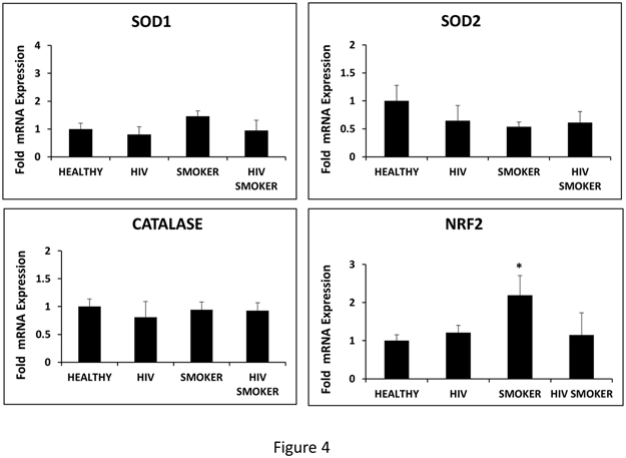
Determination of antioxidant gene expression in HIV negative non-smokers (HEALTHY), HIV positive non-smokers (HIV), HIV negative smokers (SMOKER), and HIV positive smokers (HIV SMOKER) groups. The mRNA expressions of SOD1, SOD2, catalase, and Nrf2 were measured by qRTPCR. A one way-ANOVA was employed to calculate the p value (* represent p≤0.05) with respect to HEALTHY in all the groups.

The expression of antioxidant enzymes (AOEs) are generally induced to combat increased oxidative stress triggered by an agent. Therefore we measured the levels of antioxidant genes; SOD1, SOD2, catalase, and Nrf2 in our four cohorts. Our results demonstrated that the levels of mRNA of the most antioxidant genes are not altered in HIV positive and/or smoker groups compared to HIV negative non-smokers (Fig 4). However, we found a 2-fold increase in the level of Nrf2 in HIV negative smokers compared to the HIV negative non-smoker. In general, a lack of induction of the most AOEs suggest their inability to counterbalance oxidative stress generated by smoking and HIV infection.

### Effect of smoking and HIV on the levels of CYP enzymes in monocytes

**Fig 5:**
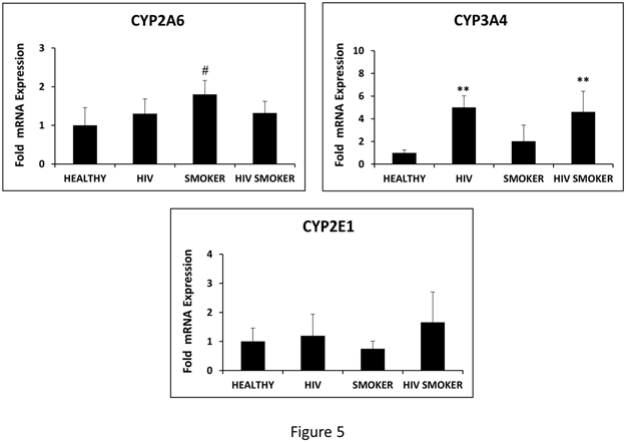
Determination of CYP levels in HIV negative non-smokers (HEALTHY), HIV positive non-smokers (HIV), HIV negative smokers (SMOKER), and HIV positive smokers (HIV SMOKER) groups. The expression levels of CYP2A6, CYP3A4, and CYP2E1 mRNA were determined by qRTPCR in all the groups. A one way-ANOVA was employed to calculate the p value (** represent p≤0.01; # represents p≤0.1, borderline significance) with respect to HEALTHY in all the groups.

CYP enzymes play important role in generating oxidative stress by generating superoxide and peroxide as a result of P450-mediated reaction cycle. Therefore, we measured the levels of important CYP enzymes (CYP2A6, CYP2E1, and CYP3A4), which are known to produce oxidative stress by metabolizing endogenous compounds or xenobiotics such as tobacco constituents and marketed drugs. As expected, the results showed that the levels of CYP2A6 mRNA is increased (p = 0.07; borderline significance) in HIV negative smokers (1.5-fold) compared to HIV negative non-smokers (Fig 5). However, the level of CYP2A6 mRNA was only marginally increased in HIV positive non-smokers and HIV positive smokers compared to HIV negative non-smokers. Interestingly, the level of CYP3A4 was increased in HIV positive non-smokers and HIV positive smokers by approximately 5-fold compared to HIV negative non-smokers (Fig 5). However, its level was not significantly increased in the HIV negative smokers. As expected, there was no significant increase in the level of CYP2E1 in HIV positive and/or smoker cohorts, suggesting that CYP2E1, which is induced by alcohol, does not play role in inducing oxidative stress in these cohorts.

## Discussion

Nicotine/tobacco smoking has shown to increase HIV replication in *in vitro* studies [2,4]. *In vitro* studies have also shown an association between oxidative stress and HIV replication [9,10,13]. However, there is no clear evidence from the clinical samples of HIV-infected smokers, especially in ART-naïve patients. Similarly, to date, there is no clinical evidence of the involvement of CYP and oxidative stress pathways in HIV and/or smoking in HIV-infected individuals. We have recently shown the involvement of CYP enzymes in nicotine metabolism and oxidative stress in *in vitro* in HIV monocyte and astrocyte model systems. In the current study, we used clinical samples from HIV-infected smokers to establish possible involvement of CYP and oxidative stress pathways in smoking-mediated HIV pathogenesis. This is the first clinical evidence of an increase in viral load in HIV-infected smokers, as well as, possible involvement of CYP and oxidative stress pathways in HIV positive smokers and non-smokers. In addition, in HIV-infected mature macrophages, HIV replication was found to be significantly higher in CSC treated macrophages compared to control cells further supporting the effects of smoking on HIV pathogenesis.

Several studies have shown that mild-to-moderate smoking leads to increased CD4 cell count and CD4/CD8 ratio [35]. In contrast, another study reported that heavy smoking caused a decline in CD4 cell count [36]. Our findings are generally consistent with the former literature in which smoking showed an increase in the CD4 counts. Our study also suggest an increase in HIV replication by tobacco smoking, which is consistent with previous *in vitro* findings [2,3]. However, this is the first report on clinical samples obtained from HIV-infected smokers. An earlier study in HIV-seropositive women has shown an association of cigarette smoking with viral immune and cognitive function [37]. However, the underlying mechanism of the effects of smoking on viral immune, cognitive function, and HIV pathogenesis is largely unknown.

HIV infection is known to increase the expression of pro-inflammatory cytokines including IL-1, IL-6, RANTES, MCP-1 and TNF-α [38,39]. In contrast, our results did not show an increase in the levels of these cytokines in HIV positive cohorts, rather there were significant decrease in the levels of IL-8 and MCP-1. There is a report demonstrating that the levels of TNF-α, IL-1β and IL-8 are significantly higher in asymptomatic HIV-infected African women than women living with AIDS, suggesting the role of cytokines in early phase of HIV infection [40]. Thus, it is likely that unaltered/decrease in cytokines levels in our study is because the HIV-infected patients had in many cases already progressed to full-blown AIDS. Likewise, cigarette smoking is associated with an increased production of pro-inflammatory cytokines, especially IL-1β and IL-1RA in bronchoalveolar lavage [41], and IL-6, IL-1β and TNF-α in the serum [42,43]. In contrast, in our study, smoking was not associated with an increase in the levels of pro-inflammatory cytokines, except RANTES, rather it showed a decrease in the levels of IL-8 and MCP-1.

Tobacco smoking has been shown to affect cytokine network balance and there by alter the host immune responses in the pathogenesis of both periodontal disease and cardiovascular disease. For instance, both nicotine and cigarette smoke condensate treatment to human endothelial cells had significantly reduced MCP-1 levels in those cells compared to control [44]. Similarly, a study conducted in smokers with computed tomography (CT) detected emphysema and without airway obstruction had demonstrated decreased plasma levels of cytokines including EGF, IL-8, IL-15, and IL-1RA than the smokers without CT detected emphysema [45]. In contrast, patients with COPD usually exhibit higher IL-8 levels. Therefore these studies suggests that the plasma cytokine profiles of smokers widely vary in individuals with either emphysema or COPD. This also suggests that smoking could differentially affect the cytokine levels based on their respiratory disease conditions which may be responsible for the decreased levels of cytokines observed in our smokers and HIV positive smokers. Moreover, it is well known that HIV-1 infection alters the levels of cytokine production in vitro as well as in vivo [46]. Nonetheless, there is no report till date about correlation between smoking associated increased viral load and cytokine levels. Therefore an extensive longitudinal study that monitors the clinical status of smokers can provide us with a better understanding of the relation between smoking prompted viral load and their cytokine profiles. In general, we acknowledge that our data show contrasting findings, which needs to be investigated further. However, it can be noted that the population demography in our study is different from other studies in terms of strict exclusion criteria of the subjects (non-HIV medication as well as use of other substances of abuse).

An increase in the oxidative stress in both plasma and monocytes in smokers and HIV-infected individuals is consistent with the previous reports, especially with monocytes [9,10,13,47]. However, this is the first evidence of increased oxidative DNA damage in the plasma and monocytes using clinical samples from HIV-infected smokers. It is noteworthy that while a previous report has shown higher serum 8-OHdG levels in males versus females [48], no gender difference in 8-OHdG level was observed in our study, at least in HIV negative non-smokers and HIV positive smokers. Further, a relatively higher levels of oxidative DNA damage in plasma compared with monocyte samples in all the groups suggests that there may be increased oxidative stress in other tissues draining into plasma. While the DNA damage in plasma may be as a result of ROS generated by liver and other blood cells including monocytes, DNA damage in monocytes is expected to entirely come from increased ROS in monocytes.

An optimal level of ROS is required for cellular functions. However, when the level of ROS is elevated, defense mechanism may counterbalance the increased ROS to protect the cells. As one of the major defense mechanism, the Nrf2 signaling pathway is activated, which results in the transcription of a myriad of AOEs such as SOD1 and catalase to protect the cells from oxidative insult [49]. However, if the ROS reaches a threshold level, the AOEs pathway is compromised leading to cellular death. On the other hand, a defect in the defense system may cause either a decrease or no change in the levels of these AOEs leading to increased level of ROS. Since there is no significant increase in the levels of AOEs determined in smokers, we suggest that the levels of ROS has reached the threshold level, or the defense system through the Nrf2 pathway may be compromised in these individuals.

The role of oxidative stress as a result of production of ROS, including nitric oxides has been implicated in HIV pathogenesis [9–11,50,51]. Similarly, previous literature [2–4,16,18,19] and our current findings demonstrate that smoking and oxidative stress both are independently associated with increased HIV replication. Thus, we suggest that smoking-mediated oxidative stress may be responsible for increased HIV replication. Although several mechanisms have been implicated in the production of oxidative stress in the HIV systems, smoking mediated production of ROS through CYP pathway may play an important role in HIV pathogenesis. Since CYP-mediated metabolism of tobacco constituents is known to produce reactive oxygen species in the liver [52], it is reasonable to conclude that CYP-mediated pathways contributes to the DNA damage in both plasma and monocytes. Our results relatively low levels of DNA damage in monocytes suggest that CYP-mediated metabolic pathway also exists in monocytes. Our speculation is consistent with our earlier observation that although monocytes contain relatively low levels of CYP enzymes compared to the liver, the relative level of CYP2A6 in monocytes is much higher that other CYP enzymes [24]. Therefore, we suggest that although CYP2A6 in smokers and HIV positive non-smokers are not induced, the basal level of CYP2A6 is sufficient to metabolize tobacco constituents and endogenous compounds and produce ROS. Furthermore, our observation is consistent with the recent finding that CYP2A6 mediated metabolism of nicotine produces ROS in monocytes and astrocytes [18,19], which may be responsible for increased DNA damage in monocytes. Another recent study from our laboratory, which demonstrated an increased metabolism of nicotine in HIV positive smokers compared to HIV negative smokers, is also consistent with this observation [53]. It is also possible that the increased metabolism is due to increased activity of enzyme rather than the actual protein level. An increase in enzyme activity through substrate-mediated enhance stability of enzyme is known in the P450 system [54].

The CYP pathway is also known to produce oxidative stress through the metabolism of endogenous compounds and xenobiotics such as marketed drugs [55]. The major drug-metabolizing enzyme CYP3A4, which is also known to metabolize many endogenous compounds and is present at relatively much higher level than other CYP enzymes in the liver, is expected to produce oxidative stress. A significant increase in the level of CYP3A4 in HIV-infected non-smokers as well as in HIV-infected smokers suggest an important role of CYP3A4 in HIV pathogenesis. It is possible that induction of CYP3A4 by HIV pathogenesis increases the metabolism of many endogenous compounds as well as xenobiotics, which in turn leads to increased oxidative stress. Since oxidative stress is also linked with HIV replication, the induction of CYP3A4 would further increase HIV replication. In addition to its role in oxidative stress, CYP3A4 may play significant role in HIV patients who are receiving ART, especially non-nucleoside reverse transcriptase inhibitor (NNRTIs), protease inhibitors (PIs), and more recently integrase inhibitors. CYP3A4 metabolizes majority of NNRTIs, integrase inhibitors, and all the PIs, which are an essential component of ART [23]. Thus, increased level of CYP3A4 in HIV positive smokers and HIV positive non-smokers would increase the metabolism of NNRTIs, PIs, and integrase inhibitors in these individuals. An increased metabolism of ART would subsequently decrease the bioavailability of these ARTs ultimately leading to decreased response to these drugs and increased toxicity. Therefore, further investigation to clarify the role of CYP3A4 in HIV-infected individuals over time is necessary.

In conclusion, the current study suggests that mild-to-moderate smoking increases viral load in HIV-infected individuals, which was further confirmed using in vitro increased viral replication by CSC in HIV-infected macrophages. Furthermore, our study suggests that smoking and HIV independently increase oxidative stress in the plasma as well as in monocytes. An increase in oxidative stress could be as a result of both, CYP-mediated hepatic metabolism of smoking constituents and/or endogenous compounds and lack of induction of AOEs. Although we suggest a possible association of CYP and oxidative stress pathways in HIV replication, other pathways cannot be excluded. Therefore, there is a need to further investigate CYP or alternative pathways in smoking mediated HIV pathogenesis using in vitro HIV-infected macrophages as well as in an in vivo HIV-infected humanized mice model.
